# Supplementary material for: Resolving the full spectrum of human genome variation using Linked-Reads
Source: Genome Res. 2019 Apr;29(4):635–45. doi: 10.1101/gr.234443.118 (PMC6442396; doi:10.1101/gr.234443.118)
Supplement: Supplemental Material [file supp_gr.234443.118_Supplemental_longranger-2.2.zip › longranger-2.2.tar.bz2!/lr2.2-public/lib/stan/cmdstan/stan/doc/api/html/rank_8hpp_source.html]

Stan: src/stan/math/matrix/rank.hpp Source File


|  |  |
| --- | --- |
|  | Stan  2.5.0  probability, sampling & optimization |


- Main Page
- Related Pages
- Namespaces
- Classes
- Files

- File List
- File Members

All Classes Namespaces Files Functions Variables Typedefs Enumerations Enumerator Friends Macros Pages

- src
- stan
- math
- matrix

rank.hpp

Go to the documentation of this file.

1 #ifndef STAN\_\_MATH\_\_MATRIX\_\_RANK\_HPP

2 #define STAN\_\_MATH\_\_MATRIX\_\_RANK\_HPP

3

4 #include <vector>

5 #include <stan/math/matrix/Eigen.hpp>

6 #include <stan/math/error\_handling/matrix/check\_range.hpp>

7

8 namespace stan {

9  namespace math {

10

17  template <typename T>

18  inline size\_t rank(const std::vector<T> & v, int s) {

19  size\_t size = v.size();

20  check\_range(size,s,"in the function rank(v,s)",s);

21  s--;

22  size\_t count(0U);

23  T compare(v[s]);

24  for (size\_t i = 0U; i < size; ++i)

25  if (v[i]<compare) count++;

26  return count;

27  }

28

35  template <typename T, int R, int C>

36  inline size\_t rank(const Eigen::Matrix<T,R,C> & v, int s) {

37  size\_t size = v.size();

38  check\_range(size,s,"in the function rank(v,s)",s);

39  s--;

40  const T \* vv = v.data();

41  size\_t count(0U);

42  T compare(vv[s]);

43  for (size\_t i = 0U; i < size; ++i)

44  if (vv[i]<compare) count++;

45  return count;

46  }

47

48  }

49 }

50 #endif

Eigen.hpp

stan::math::size

int size(const std::vector< T > &x)

**Definition:** size.hpp:11

stan::math::check\_range

void check\_range(size\_t max, size\_t i, const char \*msg, size\_t idx)

**Definition:** check\_range.hpp:30

check\_range.hpp

stan::math::rank

size\_t rank(const std::vector< T > &v, int s)

Return the number of components of v less than v[s].

**Definition:** rank.hpp:18


---

[ Stan Home Page ]

*© 2011–2014,
Stan Development Team.*
